# Cardiac Avoidance in Breast Radiotherapy: Many Choices for a Worthwhile Objective

**DOI:** 10.3389/fonc.2014.00269

**Published:** 2014-10-07

**Authors:** Atif J. Khan, Sharad Goyal, Frank A. Vicini

**Affiliations:** ^1^Rutgers Cancer Institute of New Jersey, Robert Wood Johnson University Hospital, New Brunswick, NJ, USA; ^2^Michigan Healthcare Professionals, Farmington Hills, MI, USA

**Keywords:** prone breast, deep inspiration breath hold, accelerated partial breast irradiation, proton therapy, cardiac avoidance

In this Research Topic, Goyal and Haffty have collected a series of papers on the emerging field of cardio-oncology. Indeed, Darby et al.’s paper demonstrating an incremental 7% increase in risk of ischemic events per gray increase in mean heart dose has been a watershed moment in our efforts to improve the therapeutic ratio of adjuvant breast radiotherapy (1). While a 0.07 increase over baseline risk per Sievert may seem high, it is important to understand this relative increase in risk in the context of the absolute baseline risk. Darby and colleagues do not provide a denominator for eligible patients in the two population registries from which they drew their cases and controls. Instead, they estimate the baseline risk using data from 15 Western European nations, and in Table S12 of the Supplementary Material, go on to estimate the absolute risk increase by age 80 years in women exposed to RT at various ages and with various co-morbid risk profiles. The excess absolute risks appear to be modest at first glance. For example, for a young 40-year-old woman receiving a high mean heart dose of 10 Gy, the estimated absolute excess risk of *dying* from cardiac disease is about 1.4%. Should the same woman have at least one co-morbid risk factor, her excess risk is 2.3%. These numbers may seem small, but are certainly relevant at the population level, especially given that the mortality benefit of adjuvant radiotherapy is also modest (2). Current efforts at reducing the risks of incidental cardiac irradiation have included advanced radiotherapy techniques for cardiac avoidance such as breath hold (3), gating treatments (4), proton therapy (5), prone positioning (6), and combinations thereof such as respiratory gating in the prone position (7).

Cardiac avoidance techniques are illustrative of the general potential that technological innovations can have on human health. Going back to the very development of megavoltage machines, improvements in radiation delivery have consistently improved the therapeutic ratio in any number of settings. Recent reports have demonstrated fewer late second malignancies in children treated with proton therapy (8), lower rates of desquamation in breast cancer patients treated with IMRT (9), higher rates of local control in lung cancer patients treated with SBRT (10), and improved biochemical control in patients treated with highly conformal, high-dose radiotherapy for prostate cancer (11). Similar improvements in image-guided gynecological brachytherapy (12), IMRT in head/neck (13), GI (14), and gynecological malignancies (15), as well as intracranial SRS (16) have all demonstrated better outcomes compared with control data. Even as the calls for controlling costs become ever more constant, it is important to remember that the current excitement for a genomically driven model of cancer care has become possible only because of technological improvements in sequencing technologies. As such, continued funding, both federal and private, for technology innovations is critical and should not be relegated to lower tiers of priority.

Coming back to breast cancer patients and the cardiac risks they face from radiotherapy, one additional (seemingly obvious) point needs to be made. While we can invoke continually advancing technologies for the purposes of cardiac avoidance (17), sometimes a return to simpler solutions may be all that is needed. Many women with early-stage breast cancer are eligible for off-protocol accelerated partial breast irradiation as a standard of care option (18). As one would expect, irradiating a smaller volume of breast tissue leads to lower incidental heart doses (19). Current studies and protocols examining, for example, breath hold parameters or prone positioning often include a large contingent of women who are candidates for partial breast irradiation. One rather elegant way to avoid treating the heart is to simply not treat it.

## Conflict of Interest Statement

The authors declare that the research was conducted in the absence of any commercial or financial relationships that could be construed as a potential conflict of interest.
